# Factors associated with evidence-based practice among registered nurses in Sweden: a national cross-sectional study

**DOI:** 10.1186/1472-6963-13-165

**Published:** 2013-05-04

**Authors:** Anne-Marie Boström, Ann Rudman, Anna Ehrenberg, Jens Petter Gustavsson, Lars Wallin

**Affiliations:** 1 Division of Nursing, Department of Neurobiology, Care Sciences and Society, Karolinska Institutet, Huddinge, Sweden; 2 Department of Geriatric Medicine, Danderyd Hospital, Danderyd, Sweden; 3 Division of Psychology, Department of Clinical Neuroscience, Karolinska Institutet, Solna, Sweden; 4 Department of Health and Social Studies, Dalarna University, Falun, Sweden

**Keywords:** Cross-sectional studies, Evidence-based practice, Individual factors, Logistic models, Nurses, Organizational factors

## Abstract

**Background:**

Evidence-based practice (EBP) is emphasized to increase the quality of care and patient safety. EBP is often described as a process consisting of distinct activities including, formulating questions, searching for information, compiling the appraised information, implementing evidence, and evaluating the resulting practice. To increase registered nurses’ (RNs’) practice of EBP, variables associated with such activities need to be explored. The aim of the study was to examine individual and organizational factors associated with EBP activities among RNs 2 years post graduation.

**Methods:**

A cross-sectional design based on a national sample of RNs was used. Data were collected in 2007 from a cohort of RNs, included in the Swedish Longitudinal Analyses of Nursing Education/Employment study. The sample consisted of 1256 RNs (response rate 76%). Of these 987 RNs worked in healthcare at the time of the data collection. Data was self-reported and collected through annual postal surveys. EBP activities were measured using six single items along with instruments measuring individual and work-related variables. Data were analyzed using logistic regression models.

**Results:**

Associated factors were identified for all six EBP activities. Capability beliefs regarding EBP was a significant factor for all six activities (OR = 2.6 - 7.3). Working in the care of older people was associated with a high extent of practicing four activities (OR = 1.7 - 2.2). Supportive leadership and high collective efficacy were associated with practicing three activities (OR = 1.4 - 2.0).

**Conclusions:**

To be successful in enhancing EBP among newly graduated RNs, strategies need to incorporate both individually and organizationally directed factors.

## Background

It is 20 years since the Evidence Based Medicine Working Group published the first paper on evidence-based practice (EBP) introducing new teaching aspects in medical education [1]. Today, EBP is emphasized to increase the quality of care and patient safety in healthcare, and health professionals are expected to implement evidence into their daily clinical practice [2,3]. EBP is identified as being one of five core competencies in health professional education [3]. Nursing staff, in particular registered nurses (RNs), are the largest health professional group in all sectors of healthcare [4]. The majority of RNs work in direct care of patients; assessing patients’ needs and making decisions on nursing interventions. RNs’ practice of EBP can be assumed to have a major impact on patients’ outcomes and patient safety. Hence, there is a potential to improve quality of care and patient safety by enhancing RNs’ practice of EBP. Interventions aiming to enhance RNs’ practice of EBP need to target the factors that are important for EBP.

### Nurses’ practice of EBP

EBP is often viewed as a process consisting of distinct activities, such as formulating critical clinical questions, searching for information in databases and in other sources, appraising and compiling the collected information, and finally implementing evidence and evaluating practice performance [5]. Multiple instruments exist for measuring nurses’ practice of EBP [6-8]. Some instruments use single items for the EBP activities, while others combine the items into a summative scale. The use of multiple instruments as well as different measurement approaches makes it difficult to summarize findings from the published studies on nurses’ practice of EBP. However, a number of studies from various countries have reported that nurses practice EBP and the distinct EBP activities to a low extent [8-10].

### Factors associated with nurses’ practice of EBP

A range of individual and organizational factors associated with nurses’ practice of EBP have been explored. Nurses with a higher educational level, such as a Master’s degree or qualifications at an advanced level, have reported a higher extent or more frequent practice of EBP compared with nurses with lower qualifications [10-12]. Among hospital nurses in Israel, skills in locating various research repositories and organizational support for searching and reading professional literature were associated with evidence-based nursing practice [10]. Melnyk and colleagues [13] examined the associations between health professionals’ implementation of EBP and beliefs about the value of EBP and their confidence in implementing EBP into practice (EBP beliefs), organizational culture, group cohesion and job satisfaction in a community hospital system in the US. EBP implementation was significantly associated with EBP beliefs and organizational culture. Supportive leadership has been identified as being strongly associated with nurses’ EBP practice in several studies [14,15]. Junior clinical nurses have reported more barriers compared with senior clinical nurses in regard to accessing organizational information such as clinical guidelines and protocols, access to EBP resources, and having time for practicing EBP [16]. Thus, individual factors such as educational level, years of experience and beliefs and confidence in practicing EBP, as well as organizational factors such as supportive leadership, organizational climate and access to resources, have been demonstrated to be associated with practice of EBP.

### The Swedish LANE study

In 2002, a longitudinal prospective study, the Longitudinal Analyses of Nursing Education/Employment (LANE), was initiated in Sweden [17]. The aim was to monitor the health status and professional development of newly qualified RNs in the first years of their working life. The study included three cohorts of nursing students from all 26 Swedish universities that provide nursing undergraduate education. The LANE survey consists of a suite of instruments for measuring individual and work-related variables related to psychological and physical health, as well as rates of employee and occupational turnover, and professional development [17]. New measures for assessing the extent of practicing EBP and capability beliefs regarding EBP among nursing students and RNs have been developed and validated within the LANE survey [8,18].

Previous papers from LANE have reported on the extent of EBP and research utilization among nursing students [19] and newly graduated RNs [8,18,20,21]. Findings show that the nursing students had high beliefs regarding their capability to practice EBP, although there were differences between students regarding their capability in formulating questions, searching and compiling best knowledge [19]. In addition, newly graduated RNs reported a low extent of formulating questions, searching and compiling knowledge, and implementing knowledge into practice two years after graduation [8]. Moreover, findings from a longitudinal study showed a stable low extent of EBP with no significant changes in any of the six EBP activities over the first five years of practice [21]. In order to find ways to increase EBP, it is important to explore associated factors with newly graduated RNs’ practice of EBP. To our knowledge no studies previously published have examined individual and organizational determinants for the six different EBP activities in a sample of RNs. We hypothesized that the six distinct EBP activities require various cognitive skills of the individual nurse and supportive conditions in the organization. Thus, the aim of this study was to examine individual and organizational factors associated with EBP activities among RNs 2 years post graduation.

## Methods

### Design

The study used a cross-sectional design based on a national sample of newly graduated RNs from the LANE study [17].

### Setting and participants

For this study one cohort (EX2004) from the LANE database was used. The participants in this cohort had enrolled in the nursing program in 2002. The data was collected in 2007, two years after the RNs graduated from the nursing program (late autumn 2004). The sample for the data collection consisted of 1256 respondents (out of 1657 who constituted the cohort, response rate 76%) [17]. Only the 987 respondents who reported that they actually worked as RNs at the time of the data collection were included. The excluded respondents were mostly on leave because of nurse specialist education or childbirth. The cohort has previously been shown to be representative of the total population of RNs that graduated in Sweden in the same year [17].

### Instrument

Items and scales for measuring the practice of EBP, RNs’ individual characteristics and work contextual factors from the LANE survey were used. An overview of selected dependent and independent variables is presented in Table 1.

**Table 1:** Description of dependent and independent variables tested, response alternatives and their categorization

***1. Dependent variables***	***Categorization of response alternative***
Formulate questions to search for research-based knowledge ^a^:	high extent vs. low extent
Seek out relevant knowledge using databases ^b^:	high extent vs. low extent
Seek out relevant knowledge using other information sources ^c^:	high extent vs. low extent
Critically appraise and compile best knowledge^d^:	high extent vs. low extent
Participate in implementing research-based knowledge in practice ^e^:	high extent vs. low extent
Participate in evaluating whether practice reflects current research-based knowledge ^f^:	high extent vs. low extent
The response format for the above six items ranged from 1 “To a very low extent” to 4 “To a very high extent”. High extent consists of 3 and 4 and low extent of 1 and 2.	
***2. Independent variables categorized by individual and organizational factors***	
*Individual factors*	
Sex:	female (reference) vs. male
Age:	30 years and older (reference) vs. younger than 30 years
Previous training as a nurse aide (before studies):	no (reference) vs. yes
Further study after nursing degree: have studied and study now:	no (reference) vs. yes
Evidence-based practice capability beliefs: Evidence-based capability was measured by six items ^a-f^ following the EBP process (Cronbach’s α = 0.90). The response format ranged from 0 “*No, I can’t do that*” to 10 “*Yes, I can do that*”. High capability was characterized by a mean value >7.	low capability (reference) vs. high capability
*Organizational factors*	
Present form of employment:	temporary (reference) vs. permanent
Clinical setting:	Hospital (reference) vs. primary care or older people care or psychiatric care
Full or part time:	part time (reference) vs. full time
Work shifts:	three shifts (day, evening and night) (reference) vs. office hours (Monday – Friday) or two shifts (day and evening)
Work overtime:	once per week or less often (reference) vs. several times per week
Enough staff compared to patients’ needs:	no (reference) vs. yes
Collective efficacy^g^: Collective efficacy was measured by 3 items (Cronbach’s α = 0.71). The response format ranged from 1 “Yes, I am sure we manage that” to 11 “No, we do not manage that”. High collective efficacy was characterized by a mean value ≤2.49.	low (reference) vs. high
Role clarity^h^: Role clarity was measured using three items (Cronbach’s α = 0.74). The response format ranged from 1 “very often or always” to 5 “seldom or never”. High role clarity was characterized by a mean value ≤2.	low (reference) vs. high
Leadership^i^: Leadership was measured by six items (Cronbach’s α = 0.90). The response format ranged from 1 “very often or always” to 5 “seldom or never”. High-quality leadership was characterized by a mean value ≤2.	low (reference) vs. high
Job demands^j^: Job demands were measured using four items (Cronbach’s α = 0.75). The response format ranged from 1 “very often or always” to 5 “seldom or never”. High job demands were characterized by a mean value ≤2.	low (reference) vs. high
Control^k^: Control was measured using four items (Cronbach’s α = 0.66). The response format ranged from 1 “very often or always” to 5 “seldom or never”. High control was characterized by a mean value ≤2.	low (reference) vs. high
*Job burnout*^*l*^	
Disengagement: The disengagement scale consisted of six items (Cronbach’s α = 0.83). The response format ranged from 1 “totally disagree” to 4 “totally agree”. High disengagement was characterized by a mean value >3.	low (reference) vs. high
Exhaustion: The exhaustion scale consisted of five items (Cronbach’s α = 0.74). The response format ranged from 1 “totally disagree” to 4 “totally agree”. High exhaustion was characterized by a mean value >2.5.	low (reference) vs. high

Legends:

^a-f^ Six items measuring the process of evidence-based practice [18].

^g^ The measure of Collective Professional Efficacy in the LANE survey was constructed by the research group with inspiration from Bandura’s guide for constructing self-efficacy scales [22]. The collective efficacy aims to measure the individual members’ appraisals of the capability of their group operating as a whole [23].

^h^ Role clarity, or role ambiguity, refers to a situation where role expectations are unknown or unclear [24] and originates from the QPSNordic scale that was designed as an instrument for assessing psychological, social and organizational work conditions [25].

^i^ Leadership, the leadership measure in the LANE study, consist of items from the QPSNordic scales, i.e. three dimensions “social support from superiors”, “empowering leadership”, and “fair leadership” are used together, building a scale of leadership and support from superiors [25].

^j^ The job demands items in the QPSNordic measure the individuals’ perceptions and subjective evaluations of work demands, i.e. quantitative demands and decisional demands [24].

^k^ Control at work: the control dimension in the questionnaire refers to objective aspects or perceptions of the situation at work, e.g. the presence of freedom of choice between alternatives [25].

^l^ Symptoms of burnout were measured using the Oldenburg Burnout Inventory [26] including the two dimensions exhaustion and disengagement from work. Exhaustion concerns feeling drained of energy and disengagement involves distancing oneself from one’s work, and experiencing negative attitudes towards the work in general.

Reference =1. Number of categories indicated when >2.

### Dependent variables

Six single items measuring the respondents’ extent of practicing EBP were used (Table 1). These six items have been developed based on Sackett and colleagues’ description of the EBP process, including formulating critical clinical questions, searching for relevant knowledge in databases and other information sources such as discussing with colleagues, critically appraising and compiling best knowledge, and finally implementing evidence and evaluating practice performance [5]. The items were initiated by the question “To what extent do you perform the following tasks in your work as a nurse?” and the respondents were asked to rate their extent of practicing the EBP activities using a four-point response format (1 = to a very low extent, 2 = to a low extent, 3 = to a high extent, 4 = to a very high extent). We have examined the content validity of the items using a group of RNs with expertise in EBP [8] yielding Content Validity Indices ranging between 0.8 and 1.0, indicating good content validity [27]. In addition, professional instrument developers from the technical and language laboratory at Statistics Sweden reviewed the items. For the analysis, the 4-point response format for the six items was dichotomized into low extent (1 and 2) and high extent (3 and 4) (Table 1).

### Independent variables

A modified version of the National Health Service (NHS) staff survey framework by Michie and West [28] was used to organize the independent variables for the analysis. The NHS framework was originally developed for examining work contextual factors’ influence on patient outcomes. The framework has previously been used in the LANE study to identify individual, work contextual and educational determinants of research utilization in RNs [20]. The NHS framework describes the elements in the organization, such as Work context, Management of people and performance, Individual perceptions of work, Psychological consequences for staff, Staff behavior and experiences, and Patient outcomes. The framework with its elements can be seen as indicating a ‘quasi-causality’, i.e. it illustrates a process where the elements are assumed to influence each other in the direction from context to performance. The work context is hypothesized to influence the management within the context, which in turn is hypothesized to influence individuals’ perceptions of their work context. This in turn is hypothesized to result in psychological consequences at work for the employee and, ultimately, as a final outcome, to have an impact on staff performance. In the present study, the analytic schedule consists of individual and organizational variables, and the final outcomes are the six EBP activities. Table 1 displays the independent variables, definitions, response alternatives and how the response alternatives were dichotomized for the analysis.

### Data analysis

The data were analyzed using the SPSS version 18.0 for Windows (SPSS Inc., Chicago, IL, USA). The response alternatives for the various items and scales from the LANE survey were dichotomized. Detailed information is presented in Table 1. Internal consistency for the summative scales was analyzed using Cronbach’s alpha and values are presented in Table 1. Descriptive statistics were used for frequencies and distributions. Missing data for each of the items varied between 0 and 16 (0–1.6%). A three step procedure was applied to identify variables associated with the six EBP activities. The analyses were performed as follows:

Step 1: Bivariate analyses; Spearman’s rank correlation coefficient test was used to examine the bivariate correlations between the six EBP activities and the independent variables.

Step 2: Selection of variables to be included in the final logistic regression models was done by examining multicollinearity among independent variables, with the purpose of excluding variables with strong correlation (rho-values >0.85) [29]. Furthermore, when the LANE cohort was formed, the eligible students were nested within 26 different educational institutions. The possible impact on the practice of EBP in work life of this nested educational structure was estimated using intraclass correlations (ICC).

Step 3: The logistic regression models (one for each EBP activity) was based on variables from step 2 that were entered into two blocks. The individual variables were entered solely in the first block, and in the second block the organizational variables were included, resulting in a final model. Odds Ratio (OR) was calculated, and the contribution from each block was evaluated with significance tests.

### Ethical considerations

The nursing students received oral and written information about the study, including details about confidentiality in handling of the data. They were further informed about the voluntary nature of their participation, including that they could terminate their participation at any time. Written informed consent was obtained from each participant. The LANE study was approved by the Regional Research Ethics Committee at Karolinska Institutet, Stockholm, Sweden (No. 01–045) and the Regional Ethical Review Board in Stockholm, Sweden (No. 04–587).

## Results

### Description of the sample and the extent of practicing EBP activities

In Table 2, descriptive statistics for the sample of 987 RNs are presented. Overall, the majority of the RNs were women. The mean age was 33 years, and 44 % were 30 or older. The majority worked in hospitals, and only a few in primary healthcare. Nearly two thirds scored high (>7) on EBP capability. Descriptive statistics for the remaining independent variables are presented in Table 2.

**Table 2:** Descriptive statistics on dependent and independent variables (n = 987)

**Variables**	**Response categories**	**n**	**%**
**Dependent variables**			
Formulate questions	High extent	183	19
Search databases	High extent	190	19
Search other sources	High extent	545	56
Compile information	High extent	297	31
Implement knowledge	High extent	296	30
Evaluate practice	High extent	336	34
**Independent variables**			
**Individual factors**			
Sex	Men	108	11
	Women	874	89
Age	Born 1972 or earlier	430	44
	Born 1973 or later	554	56
Previous nurse aide training	Yes	462	47
	No	516	53
Further study after nursing degree	Have studied/Study now	130	13
	No	845	87
EBP capability	High	619	63
	Low	360	37
**Organizational factors**			
Present form of employment	Permanent	566	57
	Temporary	421	43
Clinical setting	Hospital	723	74
	Primary care	42	4
	Care of older people	118	12
	Psychiatric care	101	10
Full or part time	Full time	668	68
	Part time	312	32
Work shifts (Three shifts)	Daytime	114	12
	Day and evening shifts (no nights)	466	48
	Three shifts (incl nights)	395	40
Work overtime	Several times per week	204	21
	About once a week or less often	728	79
Enough staff compared to patients’ need for care	Yes	385	39
	No	598	61
Collective efficacy	High	492	50
	Low	490	50
Role clarity	High	713	73
	Low	269	27
Leadership	High	287	29
	Low	695	71
Work demands	High	307	31
	Low	675	69
Control	High	77	8
	Low	904	92
Disengagement	High	140	14
	Low	841	86
Exhaustion	High	461	47
	Low	520	53

A variation in the RNs’ reported practice of the EBP activities was identified (Table 2). Nineteen percent reported that they formulated questions and searched in databases. Fifty-three percent searched for information using other sources. About one third of the RNs reported that they compiled information (31%), implemented evidence (30%) and evaluated whether clinical practice corresponded to current knowledge (34%).

### Factors associated with the six EBP activities

The correlations between the independent variables were scrutinized (data not shown) and no substantial correlations were found among the variables (the highest was 0.36). Therefore, all independent variables were included in the regression models for each of the six EBP activities. The ICCs were generally close to 0 ranging between 0.003 and 0.026 for the six different items two years post graduation. Thus, these near zero effects of nesting data reflect that no further control for the impact of educational institutions was needed when computing the regression models.

Associated factors were identified for each of the six EBP activities. The findings for the three EBP activities – formulating questions, searching databases and searching other knowledge sources – are presented in detail in Table 3, and the findings for the subsequent three EBP activities – compiling information, implementing evidence and evaluating practice – are presented in Table 4.

**Table 3:** Logistic regression analysis: odds ratio for higher extent of 1) formulate questions, 2) search databases and 3) search other sources in associated variables categorised as individual and organizational factors

**Framework area and variables**	**Formulate questions (n = 914)**				**Search databases (n = 918)**				**Search other sources (n = 915)**			
	**OR (CI) (step1)**	**Chi-square**	**OR (CI) (step 2)**	**Chi-square**	**OR (CI) (step1)**	**Chi-square**	**OR (CI) (step 2)**	**Chi-square**	**OR (CI) (step 1)**	**Chi-square**	**OR (CI) (step 2)**	**Chi-square**
***Block 1***				∆ 93.7***				∆ 53.2***				∆ 63.8***
***Individual factors***												
Sex	1.2 (0.7;2.1)		1.2 (0.7;2.0)		1.0 (0.6;1.7)		1.0 (0.6;1.8)		1.0 (0.7;1.6)		1.1 (0.7;1.7)	
Age	1.2 (0.8;1.7)		1.2 (0.8;1.8)		1.2 (0.8;1.7)		1.2 (0.8;1.8)		1.0 (0.7;1.3)		1.1 (0.8;1.5)	
Previous nurse aide training	1.4 (0.9; 2.0)		1.3 (0.9;2.0)		1.3 (0.9;1.9)		1.3 (0.9;1.8)		1.3 (0.9;1.7)		1.2 (0.9;1.6)	
Further study after nursing degree	1.1 (0.7; 1.8)		1.0 (0.6;1.7)		1.1 (0.7;1.8)		1.1 (0.7;1.8)		1.0 (0.6;1.4)		1.0 (0.6;1.5)	
EBP capability	8.3 (4.8;14.5)		7.3 (4.1;12.9)		4.0 (2.6;6.2)		3.8 (2.4;5.9)		3.0 (2.2;3.9)		2.6 (2.0;3.5)	
***Block 2***				∆ 30.9*				∆ 10.7				∆ 39.3**
***Organisational factors***												
Present form of employment			1.1(0.8;1.7)				0.9 (0.6;1.3)				1.2 (0.9;1.6)	
Clinical setting *Hospital*												
• Primary care			0.3 (0.1;1.1)				1.0 (0.4;2.7)				1.9 (0.8;4.3)	
• Care of older people			1.0 (0.6;1.7)				1.1 (0.6;1.9)				1.7 (1.0;2.7)	
• Psychiatry care			1.5 (0.8;2.7)				1.4 (0.8;2.6)				1.0 (0.6;1.6)	
Full or part time			0.7 (0.5;1.1)				0.7 (0.5;1.1)				1.0 (0.7;1.4)	
Work shifts (Three shifts)												
• Office hours			0.9 (0.5;1.7)				0.8 (0.4;1.4)				1.4 (0.8;2.4)	
• Two shifts			0.8 (0.6;1.3)				1.0 (0.7;1.4)				1.0 (0.7;1.3)	
Work overtime			1.2 (0.7;1.9)				1.2 (0.8;1.8)				1.5 (1.0;2.1)	
Enough staff			1.0 (0.7;1.5)				1.0 (0.7;1.4)				0.9 (0.7;1.2)	
Collective efficacy			1.3 (0.9;2.0)				1.2 (0.8;1.7)				1.4 (1.0;1.9)	
Role clarity			2.0 (1.2;3.2)				1.3 (0.9;2.0)				1.0 (0.7;1.4)	
Leadership			1.3 (0.9;2.0)				1.2 (0.8;1.8)				1.5 (1.1;2.1)	
Job demands			1.5 (0.9;2.2)				1.1 (0.8;1.7)				0.9 (0.7;1.3)	
Control			1.2 (0.7;2.2)				1.2 (0.6;2.1)				1.4 (0.8;2.5)	
Disengagement			0.8 (0.5;1.5)				1.0 (0.6;1.8)				0.8 (0.5;1.1)	
Exhaustion			1.0 (0.7;1.4)				1.1 (0.8;1.6)				0.9 (0.7;1.3)	
Final model		93.7***		124.7***		53.2***		63.9***		63.8***		103.1***

* = p<0.05; *** = p<0.001.

**Table 4:** Logistic regression analysis: odds ratio for higher extent of 1) compile information, 2) implement evidence and 3) evaluate practice in associated variables categorised as individual and organizational factors

**Framework area and variables**	**Compile information (n = 913)**				**Implement evidence (n = 917)**				**Evaluate practice (n = 919)**			
	**OR (CI) (step1)**	**Chi-square**	**OR (CI) (step 2)**	**Chi-square**	**OR (CI) (step1)**	**Chi-Square**	**OR (CI) (step 2)**	**Chi-square**	**OR (CI) (step 1)**	**Chi-square**	**OR (CI) (step 2)**	**Chi-square**
***Block 1***				∆ 53.7***				∆ 61.7***				∆ 91.0***
***Individual factors***												
Sex	1.2 (0.7;1.8)		1.1 (0.7;1.8)		0.8 (0.5;1.2)		0.7 (0.4;1.2)		0.9 (0.5;1.4)		0.8 (0.5;1.4)	
Age	0.8 (0.6;1.1)		0.9 (0.7;1.3)		1.0 (0.7;1.4)		1.2 (0.8;1.7)		1.1 (0.8;1.5)		1.3 (0.9;1.8)	
Previous nurse aide training	1.1 (0.8;1.5)		1.0 (0.7;1.4)		1.5 (1.1;2.0)		1.3 (0.9;1.8)		1.6 (1.2;2.2)		1.5 (1.1;2.0)	
Further study after nursing degree	1.1 (0.7;1.6)		1.1 (0.7;1.7)		1.1 (0.7;1.6)		0.9 (0.6;1.5)		1.1 (0.7;1.6)		1.0 (0.7;1.6)	
EBP capability	3.1 (2.2;4.3)		2.7 (1.9;3.8)		3.2 (2.3;4.5)		2.6 (1.8;3.7)		4.1 (3.0;5.8)		3.4 (2.4;4.8)	
***Block 2***				∆ 27.7*				∆ 83.8***				∆ 57.9***
***Organisational factors***												
Present form of employment			1.0 (0.8;1.4)				1.2 (0.9;1.7)				1.0 (0.8;1.4)	
Clinical setting *Hospital*												
• Primary care			1.6 (0.7;3.5)				0.7 (0.3;1.8)				0.6 (0.3;1.6)	
• Care of older people			2.1 (1.3;3.3)				2.0 (1.2;3.2)				2.2 (1.4;3.6)	
• Psychiatry care			1.4 (0.8;2.4)				2.4 (1.4;4.0)				1.6 (0.9;2.7)	
Full or part time			1.2 (0.8;1.7)				1.4 (0.9;1.9)				1.3 (0.9;1.8)	
Work shifts (Three shifts)												
• Office hours			1.0 (0.6;1.8)				1.4 (0.8;2.4)				1.0 (0.6;1.8)	
• Two shifts			0.8 (0.6;1.2)				1.1 (0.8;1.6)				1.0 (0.7;1.4)	
Work overtime			1.2 (0.8;1.8)				1.5 (0.9;2.2)				1.1 (0.7:1.6)	
Enough staff			0.9 (0.6;1.3)				1.2 (0.8;1.6)				1.0 (0.7;1.4)	
Collective efficacy			1.1(0.8;1.5)				1.7 (1.2;2.4)				1.7 (1.2;2.3)	
Role clarity			1.0 (0.7;1.4)				0.9 (0.6;1.4)				1.1 (0.7;1.5)	
Leadership			1.1 (0.8;1.5)				2.0 (1.4;2.8)				1.6 (1.2;2.3)	
Job demands			1.2 (0.8;1.7)				1.5 (1.1;2.2)				1.6 (1.2;2.3)	
Control			1.9 (1.1;3.2)				1.4 (0.8;2.5)				1.3 (0.7;2.3)	
Disengagement			0.9 (0.6;1.5)				0.6 (0.4;1.1)				0.7 (0.4;1.1)	
Exhaustion			1.2 (0.9;1.6)				0.8 (0.6;1.1)				1.0 (0.8;1.4)	
Final model		53.7***		81.3***		61.7***		145.6***		91.0***		148.9***

* = p<0.05; *** = p<0.001.

EBP capability beliefs was the only significant factor for all six activities (ORs varied between 2.6 and 7.3), i.e. high capability belief was consistently associated with a higher extent of EBP activities. Working in the care of older people was associated with a high extent of practicing four EBP activities; searching other knowledge sources (OR = 1.7), compiling information (OR = 2.1), implementing evidence (OR = 2.0), and evaluating practice (OR = 2.2). Supportive leadership and high collective efficacy were associated with a high extent of three EBP activities; searching other knowledge sources (OR = 1.5 and OR = 1.4), implementing evidence (OR = 2.0 and OR = 1.7), and evaluating practice (OR = 1.6 and OR = 1.7). High job demands was associated with a high extent of two EBP activities; implementing evidence (OR = 1.5) and evaluating practice (OR = 1.6). Additionally, one individual factor (previous nurse aide training) and four organizational factors (working in psychiatric care, working overtime, high role clarity and high control) were associated with a high extent of one of the six EBP activities (Tables 3 and 4).

## Discussion

In this study we hypothesized that the EBP activities were associated with characteristics of the individual nurse and the organizational context where the nurse works. The associations between 18 independent variables (five individual and 13 organizational characteristics) and the practice of six distinct EBP activities were examined. One individual factor (EBP capability beliefs) was significantly associated with extensive practice of all EBP activities, and three organizational factors (working in the care of older people, supportive leadership and high collective efficacy) were significantly associated with more extensive practice of three or more of the six EBP activities. These findings support our hypothesis that the six EBP activities are distinct tasks, which require various cognitive skills of the individual and supportive conditions in the organization. In the following we will discuss the implications of these findings for developing strategies for enhancing newly graduated RNs’ practice of EBP.

### EBP capability beliefs

EBP capability beliefs association with a higher extent of all EBP activities, is in line with the findings of the systematic review by Godin and colleagues [30], and shows promise for future intervention studies in nursing education and in clinical practice, as capability beliefs is a modifiable factor [31]. In the field of implementation science there is a call to increase the use of theory in research as a mean to develop interventions for increasing the uptake of evidence into practice [32]. Godin and colleagues [30] synthesized studies using social cognitive theories to determine factors influencing health professionals’ behavior and behavioral change. The theory of planned behavior [33] was identified as a useful theory, and capability beliefs (or self-efficacy) was the factor that most often predicted professionals’ behavior. In nursing research, the main focus on individual factors associated with nurses’ practice of EBP and research use (which is a major component of EBP) has been on attitudes towards research and EBP [9,34]. Some researchers have used cognitive theories in implementation research, especially for exploring the concept of capability beliefs and how it links to EBP among nurses [13,18,35]. However, to develop accurate interventions or educational methods to enhance nursing students’ and nurses’ capability beliefs regarding EBP, we believe there is a need for a more complete utilization of the conceptual framework of capability beliefs. Townsend and Scanlan [36] conducted a concept analysis of self-efficacy related to nursing students in the clinical setting. They identified four defining attributes; namely belief in being capable of performing a task (confidence), ability to carry out the task (capability), ability to be successful in performing the task over time (persistence) and ability to perform in stressful situations (strength). These four attributes and their theoretical base should preferably be considered when planning interventions to enhance EBP capability beliefs among nursing students and nurses. For example, learning interventions should include mastery experiences, role modeling, social persuasion and managing stress in practicing EBP [31]. As capability beliefs is an amendable variable, we suggest that researchers conducting intervention studies using the theory of planned behavior [33] should evaluate whether changes in EBP capability beliefs will increase, sustain or decrease over a longer time period after the intervention.

### Working in the care of older people

Working in the care of older people was the organizational factor most frequently associated with RNs’ practice of EBP activities. In Sweden, the care of older people has been a responsibility of the municipalities for the last 20 years and is today based on a social model of care [37]. The RNs’ work situation in this setting differs compared with RNs in hospitals, particularly with respect to the staff skills mix, e.g. professional groups and education levels [38]. The RNs working in the care of older people are not only accountable for planning the care of the older person, they are also expected to provide leadership for nurse aides and promote quality improvement and EBP [39]. Physicians are not employed by the municipalities but consulted as general practitioners, which puts higher demands on the RNs’ leadership and accountability for the quality of care, including medical care. Thus, the role of RNs in the care of older people requires considerable medical, nursing and pedagogical competence, as well as personal life experience [40]. It is therefore uncommon for RNs to begin their nursing career in the care of older people as the self-governed working conditions are considered to require extensive experience. In our sample, only 12% of the RNs worked in the care of older people, despite many available jobs in this sector and despite the fact that salaries in this sector tend to be higher compared with other areas of healthcare. This might imply that it is predominantly highly committed RNs who begin their career in the care of older people.

Additionally, we believe that some important regulatory, organizational and financial factors may explain the finding that working in the care of older people was associated with more EBP activity. In such care, the care provider (the municipality) is required to have a Chief Nurse who is accountable for patient safety and quality improvement, whereas this is not a requirement in the healthcare provided in hospitals and primary healthcare [41]. In 2005, the Swedish government allocated more than 1 billion SEK (104 million EURO in 2005) to support the municipalities’ work with quality of care and skills development through training for nursing staff, supervisors and leadership [42]. Additional national initiatives have been implemented to support Chief Nurses, managers and staff in the care of older people, e.g. an update of clinical guidelines and the development of indicators for quality improvement [43,44]. Furthermore, an open access database has been commissioned by the National Board of Health and Welfare – the Elderly Guide – with information on ten quality indicators [45]. Together, all these national initiatives have accentuated the need for – and provided support for – EBP in the care of older people, and in particular the responsibility of RNs in these matters.

### Leadership and team capability

Leadership has repeatedly been identified as a factor associated with the uptake of EBP [14,15]. In our study, supportive leadership was associated with the three EBP activities: Searching other sources, Implementing evidence into practice, and Evaluating practice (Tables 3 and 4). These EBP activities require collaboration in the care team within the organization. The factor Collective efficacy was also associated with these three EBP activities, underscoring the importance of collaborative work. Collective efficacy measured the RNs’ perceptions of their work group’s capability to operate to accomplish good care for patients and a good work climate. It is well known that leadership style might influence a collaborative culture or climate, and establish good conditions for the team [46]. A study of hospital staff nurses revealed that those who perceived their unit-level nurse managers to be strong motivational leaders also reported more structural empowerment in their work environment, and reported more professional practice behaviors (self-efficacy) than nurses who perceived their nurse managers to be weak leaders [47].The manager has a pivotal role in setting clear and realistic goals for EBP activities [48]. Thus, nurse managers seem to have an important role in creating an organizational culture at all levels within the healthcare system to support nurses’ practice of EBP [13,14,49].

As supportive leadership has consistently been identified as a factor associated with nurses’ practice of EBP, one could ask whether or not the newly graduated RNs in this study perceived the nurse manager to be supportive. In fact, only 29% of the newly graduated RNs reported that their nurse manager was supportive (Table 2). With this in mind, two questions could be posed. First, what kind of support do newly graduated RNs need (and expect) from their managers to be able to practice EBP? Second, what capacity and competency does the nurse manager need to be able to support the RNs in practicing EBP? Previous Swedish studies report that nurse managers had positive attitudes to EBP and quality improvement, but few of them were educationally prepared in these areas (i.e., having a Master’s degree) [50,51]. Many nurse managers were trained in the 1980’s and 1990’s when research methods and nursing science were not as prominent in nursing education as is the case today. Furthermore, RNs educated during this period did not perceive subjects such as research methods, pedagogy, sociology and management as being important [52]. This might explain why supportive leadership was not associated with three of the EBP activities, namely, Formulate questions, Search databases and Compile knowledge, which all require knowledge in research methods. In addition, nurse managers need support and clear goals from their immediate superiors. In the study by Johansson and colleagues [51], nurse managers who had a superior that stressed the importance of EBP reported a significantly higher number of activities in connection with introducing and discussing research findings with staff members, and also used research findings in quality improvement to a higher extent than nurse managers with less supportive superiors. Thus, it seems crucial to focus on developing nurse managers’ skills and knowledge in EBP, and that their immediate superior in the organization provides support and clear goals for EBP for the nurse managers as well.

### Future research

We examined the associations between RNs’ practices of six distinct EBP activities with 18 independent variables and found that for the three EBP activities Searching other sources, Implementing evidence and Evaluating practice four to six associated factors were identified, mostly organizational variables. As discussed above these three EBP activities can be considered as collaborative tasks performed in an organizational context, which might explain why several associated organizational factors were identified. The remaining EBP activities Formulating questions, Searching databases and Compiling information can be performed by an individual alone, which might require more of cognitive skills and multiple thinking strategies, e.g., critical thinking, reflection, clinical reasoning and judgment. More research is needed to identify factors associated with these three EBP activities to increase RNs practice of the EBP process. In this study qualitative data was not collected. Future research will benefit by exploring the concept of variables such as role clarity, collective efficacy, leadership, and job demands on RNs’ practice of EBP.

#### Limitations

The modified version of the NHS staff survey framework by Michie and West [28] guided the identification of appropriate organizational variables. Although the focus of the NHS framework is on examining the impact of work contextual factors on patient outcomes, we found the NHS framework helpful for selecting organizational factors from the LANE survey in building a model for the statistical analysis. However, because of NHS framework’s focus on patient outcomes instead of RNs’ practice of EBP, potential organizational variables associated with practicing the distinct EBP activities might not have been identified and included in the regression analyses. A recent published systematic review of measures assessing structural, organizational, provider, patient and innovation factors affecting implementation of health innovation identified 62 different instruments [53]. Frequently assessed organizational factors in the identified instruments not assessed in this present study, were aspects of organizational culture or climate, and organizational readiness for change. These variables should be considered in future studies.

The study has some obvious strengths. It was based on a national sample of RNs with a relatively good response rate, and the sample has been found to be representative of the national population of newly graduated RNs [17]. The instruments have been validated and tested for the target group of respondents. The weakness is that all data were self-reported and that the actual frequencies of EBP activities were not measured. In this study a fairly new scale on EBP capability beliefs was used. This scale was developed by the LANE study team using the framework proposed by Bandura [22] for measuring an individual’s beliefs regarding capability to perform a certain activity, in this case EBP [18,19]. As the six items in this new scale are similarly formulated (but with different response formats) to the items measuring the extent of practicing EBP, there is a risk of producing artificial co-variance (common method bias) [54]. However, in the validation study we also identified significant associations between EBP capability beliefs and measures of research use [18], which vouches for the validity of the scale. The present study contributes with further evidence of the new EBP capability beliefs scale as it generate findings consistent with the validation study but now used with many variables in multivariate models. Although the use of a questionnaire answered by the individual nurse implies that we measure practice of EBP at the individual level – if the nurse in fact is performing the components of EBP – we do not propose that practicing EBP is a purely individual responsibility. Rather, the findings indicate that several organizational prerequisites need to be present if the individual RN should be supported to practice EBP. However, more studies using the new EBP capability beliefs scale are needed to examine the validity of the scale in other health professional groups and in healthcare organizations outside Sweden.

## Conclusions

Although EBP has been highlighted as a core competency of health professionals since 2003, the group of newly graduated RNs in our study reported a low extent of practicing EBP. There is obviously a need to develop strategies to support newly graduated RNs in order to enhance their skills and practice of EBP. Such strategies should focus on both individual and organizational factors associated with RNs’ practice of EBP. In this study, capability beliefs regarding EBP was the sole factor associated with all six EBP activities. Future research would benefit from utilizing the theoretical framework of the concept capability beliefs proposed by Bandura to develop and evaluate interventions in clinical practice in order to enhance RNs’ practice of EBP. Furthermore, strategies need to focus on leadership in healthcare, particularly the capacity and competence of managers to facilitate and support RNs in practicing EBP in the clinical setting. Working in the care of older people was associated with EBP activities, and can be interpreted as an effect of initiatives for the quality of care at a national level. These initiatives might have supported and put pressure on managers and RNs to practice EBP. Strategies for enhancing EBP in healthcare must involve the entire healthcare organization and should not be framed as a responsibility solely for front-line staff.

## Abbreviations

EBP: Evidence-based practice; LANE: The Longitudinal Analyses of Nursing Education/Employment; OR: Odds ratio; RN: Registered nurse.

## Competing interests

The author(s) declare that they have no competing interests.

## Authors’ contribution

AMB has been part of the design of the study; analysis and interpretation of data; and responsible for drafting of the article. AR has been part of the design of the study; acquisition of data, and analysis and interpretation of data; and drafting of the article. AE has been part of the design of the study; interpretation of data; and drafting of the article. JPG has been part of the design of the study; acquisition of data, and analysis and interpretation of data; and drafting of the article. LW has been part of the design of the study; interpretation of data; and drafting of the article. All authors read and approved the final manuscript.

## Pre-publication history

The pre-publication history for this paper can be accessed here:

http://www.biomedcentral.com/1472-6963/13/165/prepub
